# Cost-Effectiveness Analyses of Digital Health Technology for Improving the Uptake of Vaccination Programs: Systematic Review

**DOI:** 10.2196/45493

**Published:** 2023-05-15

**Authors:** Yingcheng Wang, Ginenus Fekadu, Joyce Hoi-sze You

**Affiliations:** 1 School of Pharmacy The Chinese University of Hong Kong Shatin China (Hong Kong)

**Keywords:** cost-effectiveness analyses, digital health technology, vaccination coverage, vaccine, systematic review

## Abstract

**Background:**

Vaccination is the most effective strategy to prevent infectious diseases, yet vaccination coverage has not reached the target level. To promote vaccination uptake, digital health interventions (DHIs) have been used in various vaccination programs.

**Objective:**

This study aimed to perform a systematic review of the cost-effectiveness analyses of DHIs for the improvement of the uptake of vaccination programs.

**Methods:**

A literature review was conducted in MEDLINE (Ovid), Embase (Ovid), APA PsycINFO (Ovid), Web of Science, Scopus, CINAHL Ultimate (EBSCOhost), Center for Review and Dissemination, and Institute for IEEE Xplore up to October 2022. Health economic evaluations that met the following inclusion criteria were included: (1) adult or pediatric vaccination programs; (2) interventions delivered through digital technology; (3) full-scale health economic analyses including cost-effectiveness, cost-utility, cost-benefit, or cost-consequence analyses; and (4) evaluations conducted by model-based or trial-based analyses. The quality of each included study was evaluated using the Consolidated Health Economic Evaluation Reporting Standards (CHEERS).

**Results:**

The systematic review included 7 studies. Four of the cost-effectiveness studies were conducted by model-based analyses, and 3 were trial-based analyses. One study reported the additional cost per quality-adjusted life years (QALYs) gained, whereas 6 studies reported the additional cost per individual vaccinated (or return case). The vaccines targeted the human papillomavirus (HPV) vaccine, influenza vaccination, measles-mumps-rubella (MMR) vaccine, and children immunization at different ages. The DHIs were delivered by television campaign, web-based decision aid, SMS text message, telephone, and computer-generated recall letters. The studies were classified as very good (n=5) and good (n=2) qualities. One study concluded that the DHI was cost-saving, and 6 studies concluded that the DHI was cost-effective.

**Conclusions:**

This study is the first systematic review on cost-effectiveness analyses of DHIs to improve vaccination uptake. All included studies have good to very good quality on study assessment and reported the DHIs to be cost-saving or cost-effective in the improvement of vaccination uptake.

## Introduction

Vaccination is one of the most effective preventive interventions for infectious diseases. To date, more than 20 life-threatening diseases can be prevented by vaccinations, reducing morbidity and mortality extensively. The World Health Organization estimated that immunization prevented 3 to 3.5 million deaths yearly from vaccine-preventable diseases worldwide [1]. It is also estimated that vaccination would avert US $1510.4 billion in 94 low- and middle-income countries between 2010 and 2030 when compared with no vaccination [2].

Although vaccination coverage is relatively high in high-income countries, the global coverage levels have not reached optimal goals [3]. The diphtheria-tetanus-pertussis global immunization coverage dropped from 86% in 2019 to 81% in 2021, with 25 million children not receiving primary vaccination [4]. The global coverage of the human papillomavirus (HPV) vaccine among girls also fell from 20% in 2019 to 15% in 2021 [4]. This phenomenon is caused by several reasons, including vaccine hesitancy around the population [5], lack of access to vaccines in some low- and middle-income countries [6], and the COVID-19 pandemic [7,8]. It is worth noting that a low vaccine uptake rate contributes to high health economic burden. A study in the United States reported the economic burden of vaccine-preventable diseases to be US $9 billion in 2015, of which the cost of unvaccinated individuals was US $7.1 billion (accounting for approximately 80% of the disease burden) [9]. Interventions to improve vaccination coverage and uptake rates are therefore highly warranted [10].

Digital health begins with the application of information and communications technology (ICT) in health care system and keeps changing with the evolution of new technology [11]. Today, digital health combines ICT with artificial intelligence, machine learning, and all other technologies that aimed to improve the health and well-being of the individuals and population [12]. The definition of digital health by US Food and Drug Administration includes mobile health, health information technology, wearable devices, telehealth and telemedicine, and personalized medicine [13]. Digital health assists in improving diagnostic accuracy and disease treatment, supporting clinical decisions, and enhancing the delivery and management of health care for individuals [13]. Digital health interventions (DHIs) have been used in various strategies for the improvement of vaccination uptake. For instance, reminder and recall are delivered by autodialer message, telephone, SMS text messaging, email, and mobile app [14]. Education for community or special groups is also provided in the platform of web-based communication, video, and television [15].

Despite increasing evidence supporting the positive impact of digital technologies on immunization coverage [14,16], there is still a lack of review on the cost-effectiveness of DHIs for vaccination uptake. The implementation of new DHIs incurs substantial costs, including costs for setup, maintenance, and management, to support the widespread application in the public [17]. Health economic evaluation provides a comprehensive framework to compare the changes in clinical and economic outcomes associated with the application of the digital health technology. The health economic evidence provides information to the health care providers and policy makers in the informed decision-making process on resource allocation of DHIs. The objective of this study is to conduct a systematic review on cost-effectiveness analyses of DHIs for improving the uptake of vaccination programs and access the quality of included studies.

## Methods

### Search Strategy

A comprehensive literature search strategy was first developed to evaluate the cost-effectiveness analyses of DHIs for improving the uptake of vaccination programs. A preliminary search was conducted to identify potentially relevant digital technology search terms, such as “social media,” “social network,” “digital intervention,” “email,” and “mobile phone.” To construct the search strategy, we combined the search terms for “vaccination, vaccine, immunization,” “digital technologies,” and “cost-effectiveness analysis.” The following databases were searched (up to October 2022): MEDLINE (Ovid), Embase (Ovid), APA PsycINFO (Ovid), Web of Science, Scopus, CINAHL Ultimate (EBSCOhost), Center for Review and Dissemination, and Institute for IEEE Xplore. In addition, to verify retrieval completeness, a manual search on the reference lists of both included studies and associated systematic reviews was performed. This systematic review has been registered on PROSPERO (registration ID: CRD42023400329). The details of search strategy performed for each database and the PRISMA (Preferred Reporting Items for Systematic Reviews and Meta-Analyses) 2020 checklist are provided in Multimedia Appendix 1.

### Inclusion and Exclusion Criteria

Full-text English-language health economic studies that met the following inclusion criteria were included: (1) adult or pediatric vaccination programs; (2) interventions delivered through digital technology; (3) full-scale health economic analyses including cost-effectiveness, cost-utility, cost-benefit, or cost-consequence analyses; and (4) evaluations conducted by model-based (including Markov model, decision tree model, discrete event simulation, and dynamic transmission model) or trial-based analyses. An article was excluded if (1) interventions did not involve digital technology; (2) vaccination was part of a multiple-aspect intervention program; (3) partial health economic evaluation; and (4) studies were presented in a format other than journal articles (such as letters, editorials, conference abstracts and posters, comments, and thesis).

### Study Selection

EndNote was used to collect and handle all retrieved records. After deleting duplicates, screening was performed separately by 2 reviewers (WY and GF). The third reviewer (JHSY) deliberated to resolve disagreements (if any) between reviewers (WY and GF). Titles and abstracts were evaluated for eligibility in the first round. In the second phase, full-text reviews were conducted to verify potentially eligible studies based on inclusion and exclusion criteria. The whole selection method adhered to the PRISMA statement on preferred reporting items for systematic reviews and meta-analyses [18,19].

### Date Extraction

Two reviewers (WY and GF) worked independently to extract data using a standardized data extraction checklist. Disagreements during data extraction were discussed to reach a consensus. The data systematically extracted from each included study were (1) general study information (title, author, publication year, and country); (2) disease and vaccine information (disease, vaccine, and population); (3) study methodology (type of cost-effectiveness analysis, type of study design, study perspective, time horizon, digital intervention, and sensitivity analysis methods); (4) model characteristics (if applicable for model-based analyses); (5) trial or study characteristics (if applicable for trial- or study-based analyses); and (6) cost-effectiveness analysis results (incremental cost and health gains, incremental quality-adjusted life years [QALYs], incremental cost-effectiveness ratios [ICERs], and influential parameters identified by sensitivity analyses).

### Study Completeness Assessment

The Consolidated Health Economic Evaluation Reporting Standards (CHEERS) 2022 checklist was used to assess the methodological completeness of all studies included in this review [20]. The CHEERS checklist contains a total of 28 items on six categories: (1) title, (2) abstract, (3) methods, (4) results, (5) discussion, and (6) other relevant information. The following grading method, used for the quality assessment in the previous systematic reviews [21,22], was adopted in the present assessment: 1 point was granted to each CHEERS item when the item-specific requirements were fully complete, 0.5 point for partially satisfied, and 0 point for not satisfied. Each included study was then categorized into 1 of 4 completeness categories: excellent (scored ≥85%), very good (scored between 70% and 84%), good (scored between 55% and 69%), and insufficient (scored <55%) [21,23]. All studies were scored independently by 2 investigators (WY and GF), and disagreement was resolved by discussion with a third investigator (JHSY).

### Data Analysis and Presentation

A flowchart was used to illustrate the screening and selection process, including the number of included and excluded studies. The descriptive data and significant cost-effectiveness findings of the included studies were summarized. The DHI for improving vaccine uptake was considered cost-effective if it was either (1) more effective and less costly than the comparator or (2) more effective at a higher cost and the ICER was lower than the willingness-to-pay (WTP) threshold. The completeness assessment of each included study was reported.

## Results

### Search Results

**Figure 1:**
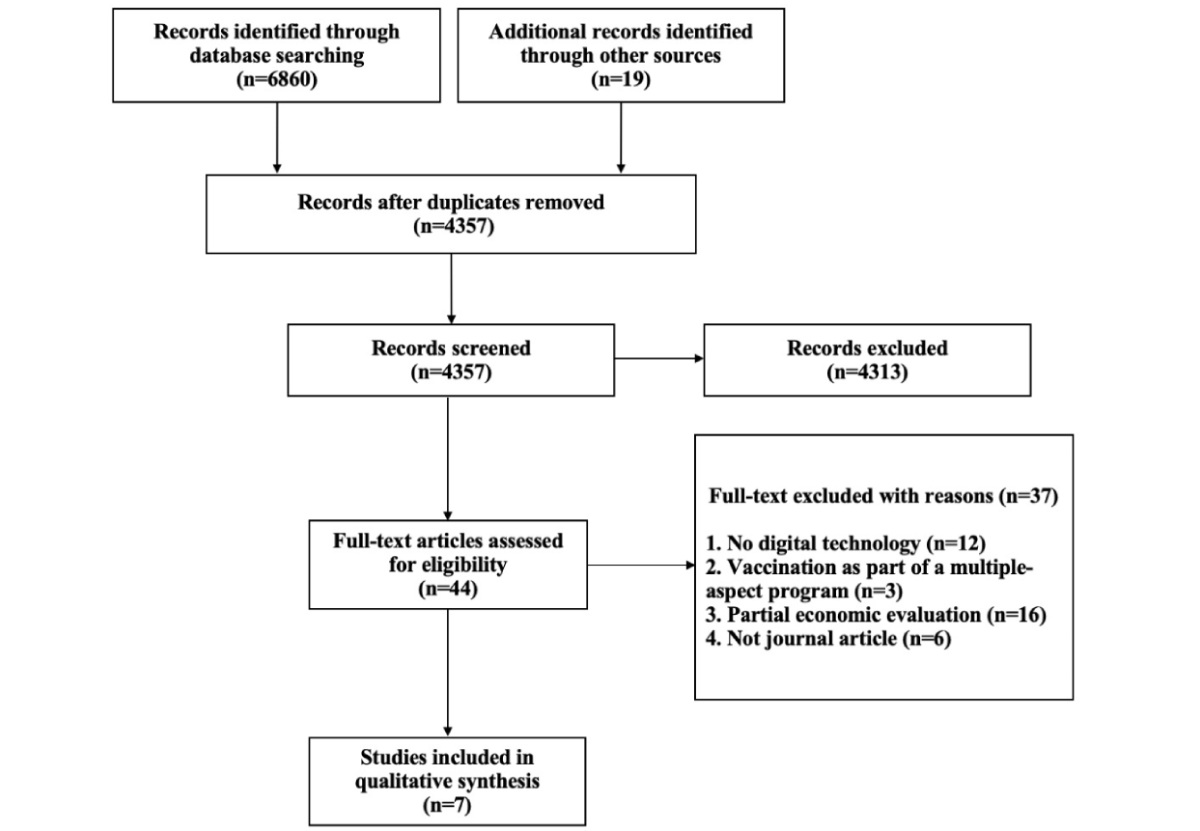
Article selection process according to the PRISMA (Preferred Reporting Items for Systematic Reviews and Meta-Analyses) statement.

The systematic review identified 6860 records in target databases and 19 records by manual searches. After the removal of duplicates, 4357 studies remained. Studies were screened by title and abstracts, leading to 44 full-text articles for eligibility. Finally, 7 studies were included in the systematic review, and the flow of the whole selection is illustrated in Figure 1.

### Study Characteristics

The characteristics of all included studies are shown in Table 1. Four studies (4/7, 57%) were model-based cost-effectiveness analyses [24-27], and 3 (3/7, 43%) were trial-based studies [28-30]. Three of 4 model-based studies (3/4, 75%) used the decision tree model [24-26], and 1 (1/4, 25%) used the dynamic transmission model [27]. Six studies reported the cost per unit of improved effectiveness (expressed as return case for immunization) [24-26,28-30] and 1 study reported the additional cost per QALY gained [27]. HPV vaccine [27], influenza vaccination [24], measles-mumps-rubella (MMR) vaccine [25,30], and vaccines for children at different ages [26,28,29] were the targeted vaccines. The DHIs delivery platforms were television-delivered education campaign [24], web-based decision aid [30], reminder by SMS text messaging [29], telephone (n=3) [26-28], and computer-generated recall letter [25]. Six of the 7 (6/7, 86%) studies targeted at children vaccination [25-30], and 1 study focused on elder individuals [24]. Three studies were conducted before 2000 [25,26,28], and the other 4 studies were conducted between 2014 and 2020 [24,27,29,30]. Most of the studies (6/7, 86%) were conducted in high-income countries including the United States [24-28] (n=5) and the United Kingdom [30] (n=1), and 1 (1/7, 14%) was conducted in a low- and middle-income country (Nigeria) [29].

The cost-effectiveness analyses were conducted from the perspective of society [24,28], government’s perspective [27,29], perspective of payer [25,26], and 1 study applied the perspective of the National Health Service (NHS) and society [30]. The dynamic transmission model used a long-term horizon (50 years) [27], whereas the other 6 studies used short-term timeframe ranging from 4 months to 1 year [24-26,28-30]. All studies have performed sensitivity analyses using probabilistic sensitivity analyses [24,27,30], 1-way sensitivity analysis [24], 2-way sensitivity analysis [25], and scenario analyses [25-29]. Except for 1 study with no funding [24], the included studies were all funded by national public health organizations (the National Institutes of Health [27], the Centers for Disease Control and Prevention [25,26,28], the National Institute for Health Research [30], and Japan International Cooperation Agency [29]).

**Table 1:** Characteristics of 7 included studies.

Authors (year); country			Targeted vaccine and population		Study type, time frame, and perspective		Interventions		Base-case and sensitivity analyses
**Web-based decision aid**									
	Tubeuf et al (2014) [30]; United Kingdom	MMR^a^ vaccine; 3- to 12-month-old individuals		Trial-based analysis; 17 months; National Health Service and societal perspective		(1) MMR web-based decision aid and usual practice, (2) MMR leaflet and usual practice, and (3) usual practice		Base-case analysis: web-based decision aid with usual practice—cost-saving and higher vaccination rate Sensitivity analysis: probability of web-based decision aid to be cost-effective was 72%-88% at WTP^b^ threshold of £0-£100 (US $0-US $124.35)	
**Television campaign**									
	Kim and Yoo (2015) [24]; United States	Influenza vaccination; Medicare individuals aged 65 years and older		Decision tree model; 1 year; societal perspective		(1) Television (nationwide) campaign and (2) no television campaign		Base-case analysis: television campaign—US $17.79 cost per additional case vaccinated Sensitivity analysis: television campaign remained cost-effective if vaccination coverage rate increased with television campaign (by at least 0.5%) for non-Hispanic White or broadcasting cost <US $14,870,000; mean ICER^c^ US $23.54 (95% CI US $14.21-US $39.37) in 10,000 Monte-Carlo simulations	
**SMS text messaging**									
	Kawakatsu et al (2020) [29]; Nigeria	Childhood vaccination; younger than 12 months		Trial-based analysis; 14 weeks; government’s perspective		(1) SMS text messaging reminder (2) no intervention		Base-case analysis: US $7.9 cost per additional return case Sensitivity analysis: two scenario analyses (excluded appointments and used the inverse-probability weighted method) found effectiveness results to be robust	
**Recall and reminder by telephone/computer**									
	Lieu et al (1997) [25]; United States	MMR vaccine; 20-month-old individuals		Decision tree model; 4 months; payer’s perspective		(1) Computer-generated recall letters and (2) no intervention		Base-case analysis: US $4.04 cost per additional child immunized Sensitivity analysis: influential factors were effectiveness of recall letters and the baseline population coverage rate Scenario analyses: US $2.14 per additional child immunized using telephone autodialer compared with no intervention	
	Lieu et al (1998) [26]; United States	Childhood immunization; 20-month-old individuals		Decision tree model; 4 months; payer’s perspective		(1) Automated telephone message alone, (2) letter alone, (3) letter followed by an automated telephone message, and (4) no intervention		Base-case analysis (vs no intervention) cost per additional child immunized: letter followed by automated telephone message: US $7.00 Automated telephone message alone: US $9.80; letter alone: US $10.50 Sensitivity analysis: cost per child immunized in automated telephone message alone decreased from US $9.8 to US $2.20 when lower cost per telephone message was applied	
	Franzini et al (2000) [28]; United States	Childhood immunization; <1 year of age		Trial-based analysis; 1 year; societal perspective		(1) Computer-autodialer reminder, (2) manual postcard mail, and (3) no intervention		Base-case analysis (vs no intervention): cost per return visit—computer autodialer: US $3.48; manual mail: US $9.52. Cost per return immunization: computer autodialer: US $4.06; manual mail: US $12.82 Sensitivity analyses: influential factors were effectiveness of computer-autodialer in return visit and immunization and start-up costs of computer-autodialer system	
	Spencer et al (2020) [27]; United States	HPV^d^ vaccine; 11-17 years old		Dynamic simulation model, 50 years; government’s perspective		(1) Reminder and recall (phone call, email, text, or mailing), (2) QI^e^ visit, and (3) school-located vaccination		Base-case analysis (vs no intervention): QI visits: US $1538/QALY^f^ reminder and recall: US $13,183/QALY; school-located vaccination: US $14,871/QALY; (WTP=US $50,000/QALY) Sensitivity analysis: Probability of being cost-effective was 83% for school-located vaccination, 12% for reminder and recall, and 5% for QI visit at a WTP of US $50,000 per QALY gained	

^a^MMR: measles-mumps-rubella.

^b^WTP: willingness-to-pay.

^c^ICER: incremental cost-effectiveness ratios.

^d^HPV: human papillomavirus.

^e^QI: quality improvement.

^f^QALY: quality-adjusted life years.

### Study Quality

The CHEERS 2022 checklist items and detailed quality assessment for each included study are listed in Multimedia Appendix 2. There were 0 (0%), 5 (71.4%), 2 (28.6%), and 0 (0%) studies classified as excellent, very good, good, and insufficient, respectively. The mean CHEERS score was 19.7 (SD 2.16, range 16.5-23; full score 28). Overall, 12 items were fulfilled by all studies (100%): (1) background and objectives, (2) health economic analysis plan, (3) study population, (4) setting and location, (5) comparators, (6) selection of outcomes, (7) measurement of outcomes, (8) rationale and description of model, (9) analytics and assumptions, (10) summary of main results, (11) study findings, limitations, generalizability, and current knowledge, and (12) source of funding. There were 11 items fulfilled in ≥50% of the studies: (1) title, (2) abstract, (3) perspective, (4) time horizon, (5) discount rate, (6) valuation of outcomes, (7) measurement and valuation of resources and costs, (8) currency, price date, and conversion, (9) characterizing uncertainty, (10) study parameters, and (11) effect of uncertainty. The remaining 5 items were only fulfilled in <50% of the studies: (1) characterizing heterogeneity, (2) characterizing distributional effects, (3) approach to engagement with patients and others affected by the study, (4) effect of engagement with patients and others affected by the study, and (5) conflicts of interest.

### DHIs for Improving Uptake of Vaccination

#### Web-Based Decision Aid

A 2014 UK study reported a health economic evaluation of a web-based decision aid that assisted parents in deciding the first dose of MMR vaccine at 12 to 13 months for their children [30]. The cost-effectiveness analysis was conducted alongside a 3-arm cluster-randomized controlled trial [31]. The parents in the control group only received the usual service provided by their registered general practitioner, and the 2 intervention groups received MMR web-based decision aid plus usual practice, and MMR leaflet plus usual practice, respectively. The web-based decision aid consisted of 2 parts: provision of disease and vaccine information (measles, mumps, and rubella diseases; MMR vaccine side effects and complications) and prompt of a decision-making process. Intervention-delivering costs were estimated from the NHS’s perspective, and parent-reported expenses were considered from a societal perspective. The web-based decision aid group showed a higher MMR vaccine uptake (42/42, 100%) than the leaflet group (69/75, 92%) and the usual practice group (61/62, 98%). From the NHS’s perspective, the web-based decision aid group was cost-saving when compared with the leaflet group (by £7.17 [US $8.92]) and the usual practice group (by £9.20 [US $11.44]). The probability of web-based decision aid to be cost-effective was 72% to 88% at the WTP threshold of £0-£100 (US $0-US $124.35) per additional child vaccinated.

#### Television Campaign

A 2015 decision tree model-based study simulated the cost-effectiveness of a hypothetical educational television campaign for promoting the uptake of seasonal influenza vaccination among US Medicare older individuals (65 years and older) [24]. The television campaign, including influenza-related topics, was assumed to be advertised for 30 seconds once weekly for 17 weeks on 3 nationwide television networks (during 8 PM to 11 PM). Costs were estimated from the societal perspective, consisting of the advertisement production cost and the broadcasting cost. Compared with no television campaign, the nationwide television campaign was estimated to increase the number of vaccinated Medicare elder individuals by 335,000 with an incremental cost of US $5,960,000, resulting in an ICER of US $17.79 per additional vaccinated case. The sensitivity analysis reported that the mean ICER for 10,000 estimates was US $23.54 (95% CI, US $14.21-US $39.37), and the cost-effectiveness of television campaign remained robust to the variation of the key model parameters. The study concluded that the hypothetical nationwide television campaign (with ICER ranging from US $14.21 to US $39.37) was cost-effective (adopting a WTP threshold of US $44.39 per additional individual vaccinated [32]). In addition, the study conducted subpopulation analyses for 4 racial and ethnic groups: non-Hispanic White, non-Hispanic African American, English-speaking Hispanic, and Spanish-speaking Hispanic. The estimated ICER (US $22.27) in the English-speaking Hispanic group was the lowest, followed by US $22.47 in the non-Hispanic White group and US $30.55 in the non-Hispanic African American group. Compared with no television campaign, the television campaign was more costly but less effective in vaccination uptake in the Spanish-speaking Hispanic group.

#### SMS Text Messages

A trial-based cost-effectiveness analysis of SMS text messaging appointment reminders for children vaccination in Nigeria was reported in 2020 [29]. In Nigeria, the childhood vaccination program required 7 visits to the primary health center for 10 types of childhood vaccines during 0 to 12 months of age. The intervention group received an SMS text messaging reminder 2 days before the upcoming vaccination appointment, and the control group received no intervention. Those who did not show up on the appointment date in the intervention group would receive an additional SMS text messaging 7 days after the appointment date. The costs considered in the analysis were start-up and recurrent costs for operating the SMS text messaging system from the governmental perspective. The effectiveness was defined as the number of return cases. The SMS text messaging reminder significantly increased the children vaccination uptake by 5%, and the estimated incremental cost per return case was US $7.90 when compared with no intervention.

#### Recall and Reminder by Telephone and Computer

Four studies examined the cost-effectiveness of using telephone and computer recalls and reminders to increase vaccination uptake. Lieu et al reported 2 studies in 1997 and 1998, targeting 20-month-old children who were overdue for MMR vaccine [25] and childhood immunizations [26], respectively, in a health maintenance organization. Both studies used a 4-month decision tree model to estimate the cost-effectiveness of different DHIs to send reminders. In the cost-effectiveness analysis for MMR vaccination [25], children in the intervention group were first identified by the computerized immunization tracking system, and then received a computer-generated personalized recall letter to inform their overdue and instruct their parents to make an appointment for vaccination. A scenario analysis on telephone autodialer for reminders was conducted. The costs of operating the computer program, clerical work, postages, printing, and stationery were considered. The proportion of children who received the MMR vaccine by 24 months of age in the intervention group (82/153, 54%) was higher than the control group (47/136, 35%). The ICER per additional child immunized was US $4.04 for computer-generated recall letters. The cost-effectiveness results were sensitive to the effectiveness of recall letters. With the variation of relative effectiveness of recall letters from 1.2 to 3, the ICER decreased from US $13.46 to US $1.35 at a 90% baseline coverage rate. In scenario analysis, the ICER of the telephone autodialer (vs the control group) was US $2.14. For childhood immunization, 4 interventions were evaluated [26]: (1) a prerecorded 1-minute automated telephone message; (2) a recall letter; (3) a letter followed by an automated telephone message; and (4) no intervention. Intervention delivery costs and the set-up costs of software programming were considered in the base-case analysis. The proportion of unimmunized 20-month-olds who received an immunization by 24 months were 44% of 165 children in the automated telephone message alone group and 44% of 162 children in letters alone group, and 58% of 154 children in the letters plus automated telephone message groups. Compared with no intervention, the cost per child immunized by 24 months of age was US $7.00 using letter plus automated telephone message, US $9.80 using automated telephone message alone, and US $10.50 using letter alone. The automated telephone message strategy was sensitive to the cost per telephone message.

A US trial-based study reported the cost and cost-effectiveness of 2 recall and reminder systems (vs no intervention) for immunizations of children <1 year old in 2000 [28]. In the autodialer group, parents received a computer-automated telephone message before their return immunization appointment date. Parents received a postcard for reminding in the manual postcard (mail) group. Cost included staff, maintenance, equipment purchasing, and supplies costs, whereas the set-up costs were considered in scenario analysis. Return visits and immunizations were the effectiveness outcomes. The number of return visits per 1000 children was 930, 886, and 669 in the autodialer, mail, and control groups, respectively. The immunized cases per 1000 children in the autodialer (n=860) and mail (n=797) groups were also higher than that in the control group (n=636). The incremental cost per return visit (relative to the control group) was US $3.48 in the autodialer group, and US $9.52 in the mail group. The incremental cost per child immunized was US $4.06 in the autodialer group and US $12.82 in the mail group (compared with the control group), demonstrating that the computer-autodialer system was more cost-effective than manual postcard mailing.

A US study in 2020 reported the findings of a dynamic transmission model for the evaluation of the cost-effectiveness of 3 HPV vaccine coverage interventions in 5 million adults and children over 50 years [27]. The 3 interventions were centralized reminder and recall (for 11- to 17-year-old individuals included in the immunization information system), school-located vaccination (for 11- to 13-year-old individuals in public middle schools), and quality improvement (QI) visits (for primary care providers participating in children vaccine program). Digital technologies were used in the reminder and recall, and QI visits. The centralized reminder and recall were delivered by phone call, email, text messaging, or mailing. The QI visits prompted the primary-care providers through providing the estimated vaccination coverage and a goal for improvement [33]. The primary model outcomes were QALYs and direct intervention-related costs (from the government’s perspective). Compared with no intervention, the ICERs were US $1538/QALY, US $13183/QALY, and US $14,871/QALY in the QI visits, reminder and recall, and school-located vaccination, respectively. Comparing reminder and recall to QI visits, the ICER was US $28,289/QALY. At the threshold of US $50,000 per QALY, all 3 interventions were accepted as cost-effective.

## Discussion

### Principal Results

This systematic review included the full-scale cost-effectiveness analyses of DHIs for improving vaccination uptake and identified a small but growing number of evidence-based studies (n=7). All the included studies were found to show high level of quality (ranging good to very good) per the CHEERS checklist. The 7 included studies were published over a long time span from 1997 to 2020, and 5 (5/7, 71%) have been published since 2000 [24,27-30]. The DHIs were delivered through automated telephone calls to targeted patients in the 2 studies before 2000 [25,26]. There was increasing application of different digital technologies for improving vaccine uptake, and the cost-effectiveness was examined in the studies reported since 2000. Most studies were conducted in resource-rich countries, but one was reported in low- and middle-income countries recently in 2020 [29]. Studies with financial support were all funded by public organizations, demonstrating that the public sector has a strong interest in implementing cost-effective DHIs to improve vaccination uptake.

All studies agreed that digital technology has a cost-effective impact on vaccination uptake. One study concluded that the web-based decision aid group was cost-saving compared to the leaflet group and the usual practice group [30]. Another study, focusing on centralized reminder and recall, QI visits, and school-located vaccination, reported that the 3 interventions were all cost-effective per QALY gained [27]. The other 5 studies used cost per additional vaccinated and/or return case as outcome measures, and the results ranged from US $2.14 to US $17.79 when compared with no intervention [24-26,28,29]. However, there was no standard WTP threshold to draw a conclusion when cost per additional vaccinated or return case was used as the outcome measure. It could only be judged whether the results were within the acceptable range by comparing them with other similar studies or using the cost-effectiveness acceptability curve to express the probability of each intervention under different WTP thresholds.

The cost-effectiveness of a DHI is highly impacted by the technology cost and the change in resource usage caused by the clinical effect of technology. The intervention-delivering cost was found to be the main cost driver in all studies. Notably, the cost of DHIs was identified as the cost-effectiveness influential factor in 3 studies [24,26,28]. The included studies contained 4 model-based studies and 3 trial-based studies. For trial-based studies, the effectiveness outcomes were sourced from 1 trial with low heterogeneity, and the cost-effectiveness findings, therefore, were subject to trial-related uncertainties. All trial-based studies had conducted a sensitivity analysis to examine the robustness of results upon variation of key parameters. For model-based studies, the effectiveness parameters were sourced from multiple trials with heterogeneity, and the impact of model input uncertainties on cost-effectiveness findings was also examined in sensitivity analyses.

### Implications

Our review identified some research gaps in the health economic evaluations of DHIs for improving vaccination uptake. First, cost-effectiveness research focused on social media platforms, such as Facebook, Twitter, or YouTube, is scarce. Over the last 2 decades, the advancement of technology on the web and mobile devices has fueled the development and popularity of social media, which has highly integrated into daily lives and is ready to influence the choice preferences of the population. The feasibility and impact of Facebook in promoting vaccination uptake were recently examined in clinical trials [34,35]. The cost-effectiveness of using social media platform as a DHI to improve vaccination uptake is therefore highly warranted. Second, the included studies have primarily focused on a few vaccines (HPV, MMR, and influenza) or age-specific cohort (children immunization). Evaluation of DHIs on the uptake of other vaccines such as the COVID-19 vaccine, hepatitis vaccine, and varicella-zoster vaccine is also in demand. Third, most included studies used a relatively short time horizon (approximately 1 year), limited to the follow-up period in randomized clinical trials. Cost-effectiveness studies with a long-term time horizon are lacking to evaluate life-ling outcomes of the impact of DHI for improving vaccination uptake. Finally, the cost-effectiveness analysis conducted in middle-income or Asian countries is lacking. In these countries, smart device subscription continues to rise due to web-based penetration, which potentially promotes the development of mobile health. The health care system begins to use DHIs for a wide spectrum of disease management, from prevention to treatment, in transitional countries [36]. The effect of DHI for promoting vaccination programs has been explored in Asia countries. A randomized controlled trial evaluated the effect of messaging education video to pregnant women in China. The findings showed that video education was significantly associated with a 4.8-fold increase in varicella vaccination coverage among their newborns [37]. Educational audio capsules and voice reminders delivered by mobile phone for improving child immunization coverage were assessed in a cluster-randomized pilot trial in India. The results found that mobile phone access was one of the key determinants of mobile health uptake [38]. Cost was also identified as a primary barrier to using mobile health [36]. Health economic evidence is warranted to evaluate the impact of DHIs on vaccine uptake, and essential to inform policy makers on the acceptable cost-effective strategies for applying DHIs in middle-income or low-income countries for vaccination programs.

### Limitations

The present review included a comprehensive search of databases and sources, and the PRISMA checklist was observed during the process. There were yet some limitations in the present systematic review. The search strategy of the systematic review only included studies written in English, and some relevant studies (in non-English languages) may be missed. The included studies applied different effectiveness outcomes and therefore limited the comparison of cost-effectiveness findings between studies. Also, the included studies were conducted in countries with different health care systems and cultures. The generalizability of health economic evidence should be cautious to scenarios of similar contexts, including, but not limited to, vaccination type, target individuals, health care systems, accessibility of mobile network, and culture.

### Conclusions

This is the first systematic review on cost-effectiveness analyses of digital technologies for improving uptake of vaccination. The included 7 cost-effectiveness studies showed “good” to “very good” quality per the CHEERS checklist. All included studies reported that the DHIs were cost-saving or cost-effective for improving vaccination uptake. The paucity of cost-effectiveness studies on DHIs for vaccination uptake indicated that further long-term evaluations on social media-delivered vaccine-specific DHIs are highly warranted.

## Appendix

Multimedia Appendix 1 Detailed information of the search terms and PRISMA 2020 checklist.

Multimedia Appendix 2 CHEERS Checklist 2022 and Study Completeness Assessment.

## Abbreviations

CHEERS: Consolidated Health Economic Evaluation Reporting Standards
DHI: digital health intervention
HPV: human papillomavirus
ICER: incremental cost-effectiveness ratio
ICT: information and communications technology
MMR: measles-mumps-rubella
NHS: National Health Service
PRISMA: Preferred Reporting Items for Systematic Reviews and Meta-Analyses
QALY: quality-adjusted life year
QI: quality improvement
WTP: willingness-to-pay
