# Supplementary material for: Cost-Effectiveness Analyses of Digital Health Technology for Improving the Uptake of Vaccination Programs: Systematic Review
Source: J Med Internet Res. 2023 May 15;25:e45493. doi: 10.2196/45493 (PMC10227707; doi:10.2196/45493)
Supplement: Multimedia Appendix 2 [file jmir_v25i1e45493_app2.docx]

**Appendix 2: CHEERS Checklist 2022 and Study Completeness Assessment**

**CHEERS 2022 Checklist**

|  | **Item** | **Guidance for Reporting** |
| --- | --- | --- |
| **TITLE** | | |
| Title | 1 | Identify the study as an economic evaluation and specify the interventions being compared. |
| **ABSTRACT** | | |
| Abstract | 2 | Provide a structured summary that highlights context, key methods, results and alternative analyses. |
| **INTRODUCTION** | | |
| Background and objectives | 3 | Give the context for the study, the study question and its practical relevance for decision making in policy or practice. |
| **METHODS** | | |
| Health economic  analysis plan | 4 | Indicate whether a health economic analysis plan was developed and  where available. |
| Study population | 5 | Describe characteristics of the study population (such as age range, demographics, socioeconomic, or clinical characteristics). |
| Setting and location | 6 | Provide relevant contextual information that may influence findings. |
| Comparators | 7 | Describe the interventions or strategies being compared and why chosen. |
| Perspective | 8 | State the perspective(s) adopted by the study and why chosen. |
| Time horizon | 9 | State the time horizon for the study and why appropriate. |
| Discount rate | 10 | Report the discount rate(s) and reason chosen. |
| Selection of outcomes | 11 | Describe what outcomes were used as the measure(s) of benefit(s) and harm(s). |
| Measurement of outcomes | 12 | Describe how outcomes used to capture benefit(s) and harm(s) were measured. |
| Valuation of outcomes | 13 | Describe the population and methods used to measure and value outcomes. |
| Measurement and valuation of resources  and costs | 14 | Describe how costs were valued. |
| Currency, price date, and conversion | 15 | Report the dates of the estimated resource quantities and unit costs, plus the currency and year of conversion. |
| Rationale and  description of model | 16 | If modelling is used, describe in detail and why used. Report if the model  is publicly available and where it can be accessed. |
| Analytics and assumptions | 17 | Describe any methods for analysing or statistically transforming data, any extrapolation methods, and approaches for validating any model used. |
| Characterizing heterogeneity | 18 | Describe any methods used for estimating how the results of the study vary for sub-groups. |
| Characterizing  distributional effects | 19 | Describe how impacts are distributed across different individuals  or adjustments made to reflect priority populations. |
| Characterizing uncertainty | 20 | Describe methods to characterize any sources of uncertainty in the analysis. |
| Approach to engagement with patients and others affected by the study | 21 | Describe any approaches to engage patients or service recipients, the general public, communities, or stakeholders (e.g., clinicians or payers) in the design of the study. |
| **RESULTS** | | |
| Study parameters | 22 | Report all analytic inputs (e.g., values, ranges, references) including uncertainty or distributional assumptions. |
| Summary of main results | 23 | Report the mean values for the main categories of costs and outcomes of interest and summarise them in the most appropriate overall measure. |
| Effect of uncertainty | 24 | Describe how uncertainty about analytic judgments, inputs, or projections  affect findings. Report the effect of choice of discount rate and time horizon, if applicable. |
| Effect of engagement with patients and others affected by the study | 25 | Report on any difference patient/service recipient, general public, community, or stakeholder involvement made to the approach or findings of the study |
| **DISCUSSION** | | |
| Study findings, limitations, generalizability, and current knowledge | 26 | Report key findings, limitations, ethical or equity considerations not captured, and how these could impact patients, policy, or practice. |
| Source of funding | 27 | Describe how the study was funded and any role of the funder in the identification, design, conduct, and reporting of the analysis |
| Conflicts of interest | 28 | Report authors conflicts of interest according to journal or  International Committee of Medical Journal Editors requirements. |

Husereau D, Drummond M, Augustovski F, de Bekker-Grob E, Briggs AH, Carswell C, Caulley L, Chaiyakunapruk N, Greenberg D, Loder E, Mauskopf J, Mullins CD, Petrou S, Pwu RF, Staniszewska S; CHEERS 2022 ISPOR Good Research Practices Task Force. Consolidated Health Economic Evaluation Reporting Standards 2022 (CHEERS 2022) Statement: Updated Reporting Guidance for Health Economic Evaluations. BMJ. 2022;376:e067975.

The checklist is Open Access distributed in accordance with the terms of the Creative Commons Attribution (CC BY 4.0) license, which permits others to distribute, remix, adapt and build upon this work, for commercial use, provided the original work is properly cited. See: [http://creativecommons.org/licenses/by/4.0/.](http://creativecommons.org/licenses/by/4.0/)

**Study Completeness Assessment**

| Items | Lieu  1997 [25] | Lieu  1998 [26] | Franzini 2000 [28] | Tubeuf 2014 [30] | Kim  2015 [24] | Kawakatsu  2020 [29] | Spencer 2020 [27] | Studies fulfilled the item (%) |
| --- | --- | --- | --- | --- | --- | --- | --- | --- |
| 1. Title | N^a^ | Y^b^ | Y | Y | Y | Y | P^c^ | 78.6% |
| 2. Abstract | P | Y | P | Y | Y | P | Y | 78.6% |
| 3. Background and objectives | Y | Y | Y | Y | Y | Y | Y | 100% |
| 4. Health economic analysis plan | Y | Y | Y | Y | Y | Y | Y | 100% |
| 5. Study population | Y | Y | Y | Y | Y | Y | Y | 100% |
| 6. Setting and location | Y | Y | Y | Y | Y | Y | Y | 100% |
| 7. Comparators | Y | Y | Y | Y | Y | Y | Y | 100% |
| 8. Perspective | N | N | N | Y | Y | Y | Y | 57.1% |
| 9. Time horizon | N | N | Y | Y | Y | Y | Y | 71.4% |
| 10. Discount rate | N | N | Y | Y | N | Y | Y | 57.1% |
| 11. Selection of outcomes | Y | Y | Y | Y | Y | Y | Y | 100% |
| 12. Measurement of outcomes | Y | Y | Y | Y | Y | Y | Y | 100% |
| 13. Valuation of outcomes | Y | Y | Y | Y | Y | Y | N | 85.7% |
| 14. Measurement and valuation of resources and costs | Y | Y | Y | Y | Y | Y | P | 92.9% |
| 15. Currency, price date, and conversion | N | N | Y | Y | Y | Y | Y | 71.4% |
| 16. Rationale and description of model | Y | Y | N.A.^d^ | N.A. | Y | N.A. | Y | 100% |
| 17. Analytics and assumptions of model | Y | Y | N.A. | N.A. | Y | N.A. | Y | 100% |
| 18. Characterizing heterogeneity | N | N | N | N | Y | N | N | 14.29% |
| 19. Characterizing distributional effects | N | N | N | N | N | N | N | 0% |
| 20. Characterizing uncertainty | Y | Y | Y | N | Y | N | Y | 71.43% |
| 21. Approach to engagement with patients and others affected by the study | N | N | N | N | N | N | N | 0% |
| 22. Study parameters | N | P | Y | P | Y | N | P | 50.00 |
| 23. Summary of main results | Y | Y | Y | Y | Y | Y | Y | 100% |
| 24. Effect of uncertainty | Y | Y | Y | Y | Y | N | Y | 85.71 |
| 25. Effect of engagement with patients and others affected by the study | N | N | N | N | N | N | N | 0% |
| 26. Study findings, limitations, generalizability, and current knowledge | Y | Y | Y | Y | Y | Y | Y | 100% |
| 27. Source of funding | Y | Y | Y | Y | Y | Y | Y | 100% |
| 28. Conflicts of interest | N | N | N | Y | N | Y | Y | 42.9% |
| Total (%) | 16.5 (58.9%) | 18.5 (66.1%) | 19.5 (75.0%) | 20.5 (78.9%) | 23 (82.1%) | 18.5  (71.2%) | 21.5  (76.8%) |  |
| Quality categories^e^ | good | good | very good | very good | very good | very good | very good |  |

^a^N = No (score 0).

^b^Y = Yes (score 1).

^c^P = Partial (score 0.5).

^d^N.A. = not applicable.

^e^Quality categories: Excellent scoring, >85.0%; very good scoring, 70.0%–84.0%; good scoring, 55.0%–69.0%; insufficient scoring, <55.0%.
